# DEPRESSION PREVALENCE IS HIGHER IN DISADVANTAGED NEIGHBORHOODS

**DOI:** 10.1093/geroni/igae098.2668

**Published:** 2024-12-31

**Authors:** Jacob Mitchell, Elizabeth Pfoh, Douglas Gunzler, Michael Kenyhercz, Lyla Mourany, Paul Gunsalus, Adam Perzynski, Jarrod Dalton

**Affiliations:** Cleveland Clinic, Cleveland, Ohio, United States; Cleveland Clinic, Cleveland, Ohio, United States; CWRU, Cleveland, Ohio, United States; Cleveland Clinic, Cleveland, Ohio, United States; Cleveland Clinic, Cleveland, Ohio, United States; Cleveland Clinic, Cleveland, Ohio, United States; MetroHealth and Case Western Reserve University, Cleveland, Ohio, United States; Cleveland Clinic, Cleveland, Ohio, United States

## Abstract

Depression is associated with premature morbidity and mortality. Knowing depression prevalence across diverse neighborhoods can inform healthy aging interventions. In this retrospective study, we analyzed electronic health data for mid-life adults (40-55 years) with ≥1 primary care appointment between 2010 and 2016 at two health systems in Cuyahoga County, Ohio. Patients were categorized as having depression if they had a diagnosis within 6 months of their first visit. Patient’s addresses were associated with the block group Ohio Area Deprivation Index (ADI), a measure of disadvantage. We used multivariable logistic regression to identify the predicted probability of depression across patients living in advantaged (ADI quintile 1) versus disadvantaged (ADI quintile 5) neighborhoods. We included race, given histories of neighborhood segregation in our location. We used a three-way interaction term between sex, race, and ADI quintile. Our sample included 150,710 patients, of which 14% had a diagnosis of depression. A higher percentage of patients with depression (versus not) were female (70% versus 54%), White (63% versus 56%), and lived in ADI quintile 5 (28% versus 25%). The predicted probability of depression was lower for patients living in ADI quintile 1 versus 5: White males (0.08, 95%CI: 0.08, 0,08 versus 0.16, 95%CI: 0.15, 0.17); White females (0.17, 95%CI: 0.16, 0.17 versus 0.25, 95%CI: 0.24, 0.26), Black males (0.05, 95%CI: 0.04, 0.07 versus 0.09, 95%CI: 0.09, 0,10) and Black females (0.14, 95%CI: 0.12, 0.16 versus 0.16, 95%CI: 0.15, 0.16). Place-based, mental health-promoting interventions may be needed to promote healthy aging among mid-life adults.

